# The contribution of maternal glucose to birth weight is smaller in Uganda (sub-Saharan Africa) than in Afro-Caribbean or white ethnicity mother–child pairs from outside Africa

**DOI:** 10.1136/bmjgh-2025-019569

**Published:** 2026-03-09

**Authors:** Wisdom P Nakanga, Isaac Sekitoleko, Rob C Andrews, Alice E Hughes, Salome Tino, Rachel M Freathy, Beverley M Shields, William L Lowe, Angus Jones, Andrew T Hattersley, Moffat J Nyirenda

**Affiliations:** 1 Medical School, MRC/UVRI and LSHTM Uganda Research Unit, Entebbe, Uganda; 2 University of Exeter, Exeter, UK; 3 MRC/UVRI and LSHTM Uganda Research Unit, Entebbe, Uganda; 4 Medical School, University of Exeter, Exeter, UK; 5 Department of Clinical and Biomedical Sciences, University of Exeter, Exeter, UK; 6 Institute of Biomedical and Clinical Science, University of Exeter, Exeter, UK; 7 Northwestern University Feinberg School of Medicine, Chicago, Illinois, USA; 8 NCD Epidemiology, London School of Hygiene & Tropical Medicine, London, UK

**Keywords:** Global Health, Maternal health, Diabetes

## Abstract

**Introduction:**

Glucose is a major determinant of fetal growth, but its relative contribution in different ethnic groups or populations is not fully understood. The Hyperglycaemia and Adverse Pregnancy Outcome (HAPO) Study established a relationship between glucose and birth weight in multiple ethnic groups. However, the HAPO Study did not include any cohorts from sub-Saharan Africa (SSA), where 17% of the world population lives. This study aims to address this in a cohort study from Uganda.

**Methods:**

We compared the relationship between oral glucose tolerance test measures and fetal outcomes in participants from Uganda (n=2544), Afro-Caribbean participants in HAPO (n=1224) and white participants in HAPO (n=7679). We used multivariable linear regression to assess the correlation between birth weight adjusted for gestational age and sex with maternal glucose concentration. Logistic regression was used to determine the association of large for gestational age (LGA) (defined as birthweight >90th percentile) with maternal fasting glucose.

**Findings:**

The contribution of maternal fasting glucose to birth weight was substantially lower in Uganda than in other settings: β-coefficient (95% CI) 104 (58.6 to 149) g/mmol/L in Uganda, 203 (137 to 270) g/mmol/L HAPO-Afro-Caribbean (AFC) and 239 (214 to 265) g/mmol/L HAPO-white. Likewise, the risk of LGA with higher fasting glucose was smaller in Uganda compared with the HAPO cohorts (adjusted OR (95% CI) 1.13 (1.00 to 1.29) in Uganda, 1.38 (1.15 to 1.66) HAPO-AFC, and 1.57 (1.46 to 1.69) HAPO-white. The contribution of glycaemia was similar using 1-hour and 2-hour post-glucose load concentrations in place of fasting glucose.

**Interpretation:**

The contribution of maternal glucose to birth weight and the risk of LGA at a given level of hyperglycaemia is substantially lower in SSA than in populations in the HAPO study. These data support the need for locally derived glycaemia cut-offs to identify women at risk of adverse pregnancy outcomes in SSA.

**WHAT IS ALREADY KNOWN ON THIS TOPIC**

- The international diagnostic criteria for gestational diabetes mellitus (GDM) are based on the Hyperglycaemia and Adverse Pregnancy Outcome (HAPO) study, which did not include any centres from sub-Saharan Africa (SSA).
- In the absence of evidence, it is assumed the impact of glycaemia is universal and so the same cut-offs as found in Europe, Asia and America are appropriate in Africa.

**WHAT THIS STUDY ADDS**

- We compared a large Ugandan birth cohort with the white and Afro-Caribbean cohorts from the HAPO Study that was used to define glycaemia cut-offs for the diagnosis of GDM.
- We show that in Uganda the impact of maternal fasting glucose on corrected birth weight is around half (104 g/mmol/L) of that seen in the HAPO-Afro-Caribbean (203 g/mmol/L) and HAPO-White participants (239 g/mmol/L).
- We also found that an increase of glucose by 1 SD in Ugandan women was associated with a smaller OR of having a large for gestational age baby than in the other populations.

**HOW THIS STUDY MIGHT AFFECT RESEARCH, PRACTICE OR POLICY**

- Our findings imply that reference cut-offs for GDM derived from studies which did not include any African populations are not appropriate for Uganda as the risk of increased fetal growth and macrosomia associated with these glucose levels is less than observed in other populations.
- This data support that locally derived glycaemia cut-offs should be used to identify women at risk of adverse pregnancy outcomes in SSA.

## Introduction

 The Hyperglycaemia and Adverse Pregnancy Outcome (HAPO) Study was a landmark study conducted to clarify the risk of adverse outcomes associated with various degrees of maternal glucose intolerance less severe than overt diabetes mellitus.^1^ This large, multinational study recruited pregnant women from nine countries (predominantly four self-reported ethnicities: White, Black, Asian and Hispanic). The study demonstrated a strong, continuous association of maternal glucose levels, measured fasting and after stimulation during a 75 g oral glucose tolerance test (OGTT) at 24–32 weeks gestation, and adverse pregnancy outcomes including large for gestational age (LGA) (birth weight above 90th percentile), caesarean section, neonatal hypoglycaemia and shoulder dystocia. No differences in the relationship between maternal glucose and pregnancy complications in the different ethnicities were reported.

The similar relationship between maternal glucose and pregnancy complications in the different ethnicities contributed to the proposal of universal cut-offs for the diagnosis of GDM. Based on the HAPO Study, the International Association of Diabetes and Pregnancy Study Groups (IADPSG) consensus panel recommended new international thresholds for diagnosing Gestational Diabetes Mellitus (GDM) that identified mothers who were at or above a 1.75-fold higher odds of having infants who were LGA due to their measured glycaemia. They therefore recommended diagnostic glucose cut-offs during an OGTT of at least one of fasting: >5.1 mmol/L, 1 hour: >10.0 mmol/L and 2 hours: >8.5 mmol/L.^2^ The WHO adopted the IADPSG guidelines in 2013.

Importantly, the HAPO Study did not recruit participants from any country in sub-Saharan Africa (SSA), which has a combined population of 1.2 billion people (17% of the world population). Maternal fasting glycaemia explains 2%–13% of the variance in birth weight in western white populations,^3 4^ but the strength of this relationship is not known in SSA. Similarly, it is unclear if the relationships between maternal glucose and adverse pregnancy outcomes in SSA are similar to those observed in high-income countries. SSA has the highest genetic heterogeneity, and other exposures prevalent in the region, such as endemic chronic infection and malnutrition, might modify this relationship.

When the IADPSG cut-offs are used, the overall prevalence of gestational diabetes in SSA is 9.6% (95% CI 7% to 12%).^5^ The risk factors for GDM are similar to those found in other settings, but the prevalence is lower.^5 6^ However, the relationships between glycaemic levels and pregnancy outcomes have not been rigorously explored in this population.

We therefore aimed to examine the relationship between maternal glycaemia and offspring birth weight and LGA, as an adverse pregnancy outcome, in Uganda. We compare these relationships with those in black and white ethnicity participants from the HAPO Study.

## Methods

### Uganda GDM study

Women attending antenatal care at five major hospitals in urban and periurban areas of central Uganda between 13 June 2018 and 31 October 2019 were screened for inclusion and exclusion criteria, shown below and invited to participate in the study. All subjects’ self-defined ethnicity was ‘black’.

All participants were aged >18 years at recruitment and between 24 and 28 weeks of gestation calculated using date of last menstrual period and/or earliest obstetric ultrasound scan where available. Women were not recruited if they were known to have diabetes prior to the current pregnancy, if they were unable to give informed consent, if they had significant medical conditions (eg, heart failure, renal disease, severe anaemia, autoimmune disease or pre-eclampsia), or if they had a multiple pregnancy.

Participants attended fasted (>9 hours) for data collection and an OGTT. Following collection of blood for fasting plasma glucose (FPG), participants consumed a 75 g liquid glucose load, with plasma collected for further glucose measurement 1 and 2 hours later.

Samples were immediately centrifuged at study sites, and plasma was stored on ice and returned immediately to the central laboratory. All samples were analysed for glucose centrally at the Medical Research Council/Uganda Virus Research Institute and London School of Hygiene & Tropical Medicine Clinical and Diagnostics Laboratory in Entebbe, Uganda, within 4 hours of collection. The plasma glucose was measured by the glucose oxidase method using the Cobas 6000 analyser (Roche/Hitachi, Tokyo, Japan).

Where local criteria for gestational diabetes were met (fasting glucose >5.8 mmol/L, and/or 2 hours >11.1 mmol/L) participants and clinicians were informed of results. These participants were excluded from further analysis. Where glucose was below these levels, the results were not made available to clinicians. Participants received obstetric and neonatal care routinely provided in their centres.

### HAPO study

The HAPO study protocol has previously been reported.^7^ Pregnant women were recruited at 15 centres in nine countries (Australia (2 centres), Barbados (1), Canada (1), China (1), Israel (2), Thailand (1), Singapore (1), UK (2) and the USA (4)). They were mainly from four self-defined ethnicities; White, non-Hispanic 46.2%, Black, non-Hispanic 11.5%, Asian 31.1%, Hispanic 8.9%, with 2.3% being other, undefined or mixed race. All subjects underwent a 75 g OGTT at 24–32 weeks of gestation between July 2000 and April 2006.

All pregnant women at a given centre were eligible to participate unless they had one or more of the following exclusion criteria^7^: age younger than 18 years, a plan to undergo delivery at another hospital, an uncertain date of last menstrual period and no ultra-sonographic estimation between 6 and 24 weeks of gestational age, inability to complete OGTT within 32 weeks of gestation, multiple pregnancy, conception using gonadotropin ovulation induction or in vitro fertilisation, glucose testing before recruitment or a diagnosis of diabetes before the current pregnancy, participation in another study that could interfere with the HAPO study, infection with the HIV or hepatitis B or C virus, previous participation in the HAPO study, or inability to converse in the language used on centre forms without the aid of an interpreter.

Detailed anthropometric measurements of the women, together with FPG, 1-hour and 2-hour glucose concentration after 75 g glucose load, were measured. Data were masked except when plasma glucose concentrations were outside the predefined values (fasting >5.8 mmol/L and/or 2 hours >11.1 mmol/L or any value <2.5 mmol/L); such patients were unmasked and removed from the main study. Otherwise, participants received obstetrical and neonatal care routinely provided in their centre. Glucose analysis was undertaken at the central laboratory using the oxidase/peroxidase method.^8^

In this analysis, we studied the populations who self-defined as ‘white’ or ‘black’. The white population was predominantly recruited from the UK, USA, Australia, Canada and Israel. The black population was predominantly recruited from Barbados (99%) and was of Afro-Caribbean descent.^9^

### Participants included in this analysis

We used similar enrolment criteria to the HAPO Study for the Uganda GDM study. We, therefore, analysed all women with singleton pregnancies without pre-existing diabetes. Individuals were only included if they had birth weight and maternal OGTT results. They were excluded if they had marked hyperglycaemia (fasting glucose >5.8 mmol/L and 2 hour glucose >11.1 mmol/L).

### Outcomes

In each cohort, we assessed (1) the association of maternal glucose concentrations with the adjusted fetal birth weight (adjusted for gestational age and sex) and (2) the association of maternal glucose concentration with the adverse outcome of LGA (defined as birth weight greater than the 90th percentile for gestational age in the individual populations).

### Statistical analyses

The main analysis was between the black Ugandan participants, white participants in HAPO and black (Afro-Caribbean) participants in HAPO. Descriptive statistics are reported for continuous (mean, SD) and categorical (number, percentage) variables, respectively. The ‘corrected birth weight’ variable was prepared by saving unstandardised residuals from a linear regression analysis of birth weight (g) against sex and gestational age. Multiple linear regressions were used to analyse associations between corrected birth weight and maternal fasting and post-load glucose with maternal glucose as continuous variables. Multiple logistic regression was used to determine the association between LGA and maternal glucose. Glucose measurements were standardised for analysis so ORs and regression coefficients represent a 1 SD increase in outcome for fasting, 1-hour and 2-hour glucose measurement. To enable comparison of slopes between the three different groups, we used an ethnicity group x glucose measurement interaction term in regression models. The final models were then adjusted for maternal age, body mass index and parity at the time of the OGTT. A sensitivity analysis was performed where we excluded women with an FPG>5.1 mmol/L (the threshold recommended by the IADSPG for diagnosis of GDM). We also compared the characteristics of those excluded because of incomplete data with those included in the analysis.

We repeated all the analyses using 1-hour and 2-hour post-load glucose concentrations for both the Uganda and HAPO cohort. Pearson product-moment correlations were used to assess associations among glucose measures within the Uganda and HAPO studies.

All analyses were performed using Stata V.16 (StataCorp) or R V.1.3.

### Role of the funding source

The funders of the study had no role in study design, data collection, data analysis, data interpretation or writing of the report.

### Patient and public involvement

The public and patients were involved in the study through the community advisory boards, where they contributed to the study design and outcome measures selection, ensuring the research directly addressed their concerns and priorities.

## Results

### Eligible subjects in the Uganda cohort

**Figure 1:**
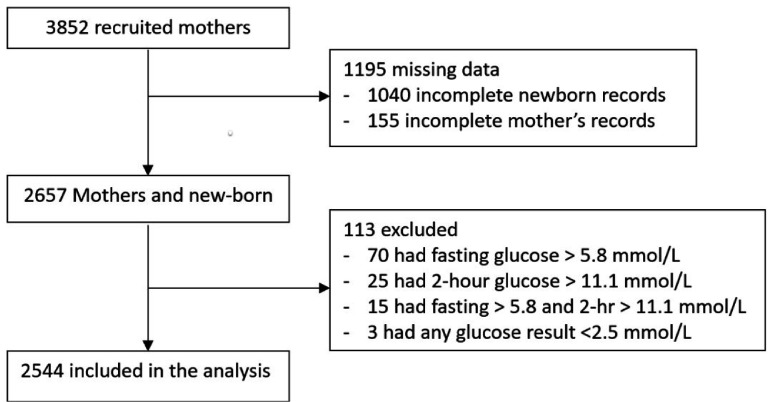
Study flow chart for the Uganda study data.

Figure 1 shows the effect of study exclusion criteria and missing data in the Uganda GDM study cohort. Of the 3852 participants recruited, 1195 could not be included in the analysis due to missing data (predominantly missing newborn records, n=1040). The participants with missing data compared with the included participants were slightly younger (25.7 vs 27.0 years) and had a slightly lower body mass index (BMI) (27.2 vs 27.8), but they had similar glycaemia at all points of the OGTT and similar blood pressure (online supplemental table 1). 123 participants were excluded because glucose values met local criteria for gestational diabetes (fasting glucose >5.8 mmol/L and 2-hour glucose >11.1 mmol/L).

### Participants

The numbers in the three cohorts after exclusions were 2544 in the Ugandan cohort, 1224 in the HAPO Afro-Caribbean cohort and 7679 in the HAPO white cohort. Characteristics of the mothers and newborns and pregnancy outcomes are summarised in table 1. All the participants in the Uganda cohort were black. White women in HAPO were the oldest, and Afro-Caribbean women in HAPO were the youngest (Uganda mean age 26.7±5.5 years, HAPO-Afro-Caribbean 25.5±5.7 years, HAPO-white 29.9±5.6 years). The mean fasting glucose was lower in the Uganda cohort than in the other cohorts (4.2±0.4 Uganda, 4.5±0.4 HAPO-Afro-Caribbean, 4.5±0.4 HAPO-white). Afro-Caribbean women in HAPO had the lightest babies, and white women in HAPO had the heaviest babies (3285±525 g, Uganda; 3227±449 g, HAPO-Afro-Caribbean; 3348±475 g HAPO-white).

**Table 1:** Characteristics of the study participants and their offspring

	Uganda N=2544	HAPO-Afro-Caribbean N=1224	HAPO-White N=7679
Maternal characteristics			
Age (years)	26.7±5.5	25.5±5.7	29.9±5.6
Body mass index (kg/m^2^)	27.6±5.0	27.8±6.0	28.1±4.5
Systolic blood pressure (mm Hg)	103±10	103±9.9	107±10
Plasma glucose (mmol/L)			
Fasting	4.2±0.4	4.5±0.4	4.5±0.4
1 hour	6.3±1.4	6.8±1.4	7.2±1.7
2 hours	5.8±1.1	6.0±1.2	5.9±1.2
Length of gestation at time of OGTT (wk)	25.9±1.2	27.1±1.8	27.8±1.7
Number of mothers parity >1 %	1775 (63.5)	355 (29.3)	2146 (28.2)
Any prenatal smoking, %	17 (0.7)	5 (0.4)	941 (12.3)
First degree relative with diabetes, %	650 (24.7)	209 (17.2)	1523 (30.9)
New-born characteristics			
Gestational age at delivery (weeks)	38.7±1.8	39.8±1.2	39.9±1.3
Birth weight (g)	3285±525	3227±449	3348±475
Male, %	1274 (50.4)	634 (51.8)	3918 (51.0)
Corrected birth weight (g)	3270±519	3228±433	3346±430

Mean±SD for continuous variables n (%) for proportions.

HAPO, Hyperglycaemia and Adverse Pregnancy Outcome; OGTT, oral glucose tolerance test.

### The contribution of maternal fasting glucose to fetal birth weight is substantially smaller in Uganda compared with Afro-Caribbean and white cohorts from the HAPO study

**Figure 2:**
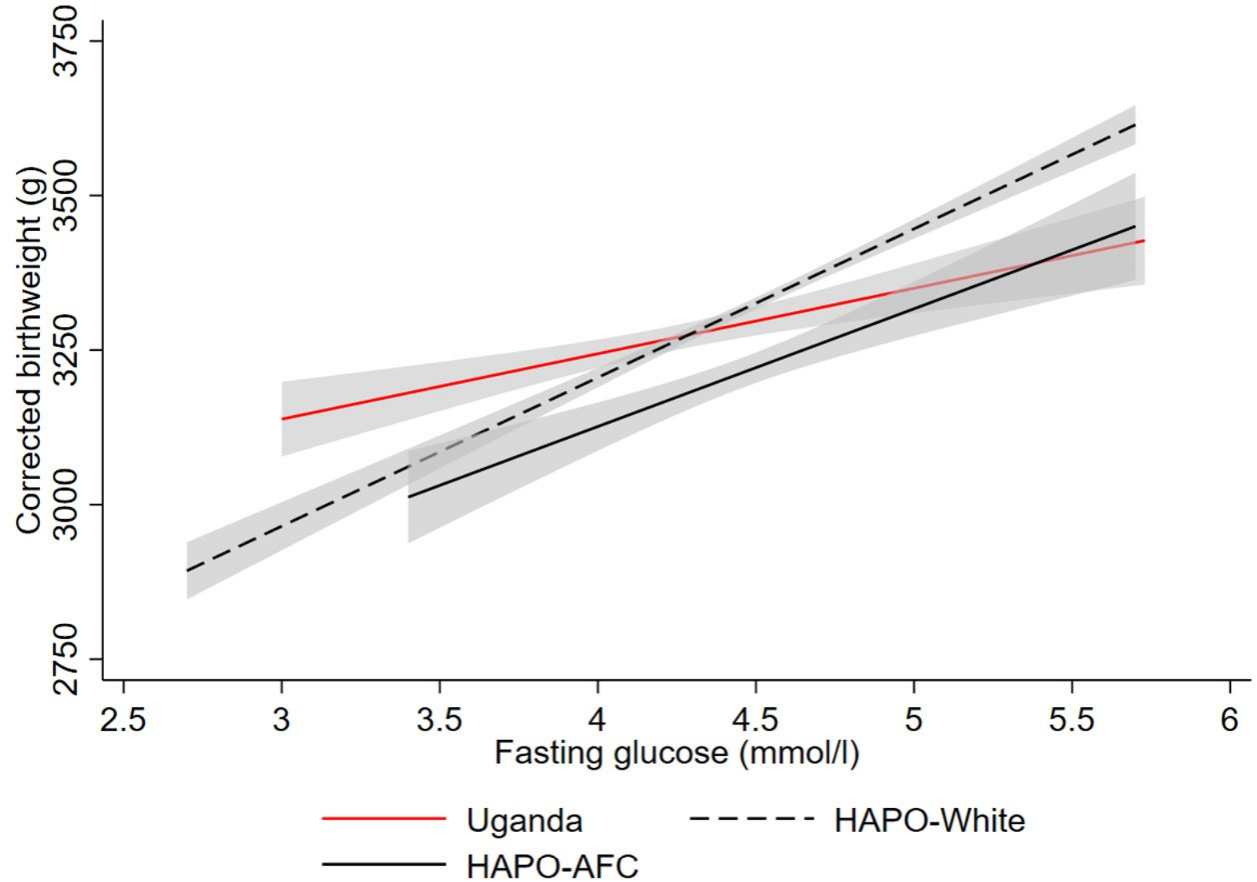
Regression lines showing the relationship between adjusted birth weight and maternal fasting glucose. Uganda (n=2544) red solid line, HAPO-Afro-Caribbean (AFC) (n=1224) black solid line and HAPO-White (n=7679) black dotted line. The shading in grey reflects the 95% CI around the regression line. Corrected birth weight was internally calculated using birth weight adjusted for gestational age and sex. HAPO, Hyperglycaemia and Adverse Pregnancy Outcome.

Figure 2 shows the association between maternal fasting glucose and the corrected offspring birth weight. There is a linear relationship between maternal fasting glucose and corrected fetal birth weight in Uganda, as was observed in the HAPO cohorts. However, the relationship was weaker in Uganda. In univariate linear regression analysis, the β-coefficients for the relationship between corrected fetal birth weight and maternal fasting glucose were lower in Uganda than in the two HAPO cohorts in unadjusted analysis (β-coefficient (95% CI) 104 (58.6 to 149) g per mmol/L in Uganda per 1SD increase in fasting glucose, 203 (137 to 270) g/mmol/L HAPO-Afro-Caribbean, and 239 (214 to 265) g/mmol/L HAPO-white (table 2)). Similarly, the variance in the birth weight explained by glucose (measured as R^2^) was markedly less in the Uganda population (0.008 Uganda vs 0.036 HAPO-Afro-Caribbean and 0.043 HAPO-white). These results were similar when adjusted for maternal age, BMI and parity, or when the HAPO study cut-off of fasting glucose >5.8 mmol/L or the IADPSG-recommended threshold of fasting glucose >5.1 mmol/L for the diagnosis of GDM was used in Uganda (online supplemental table 2).

**Table 2:** The association between corrected birth weight with maternal glycaemia and other maternal characteristics in the three populations studied

Predictor	Uganda (n=2544)			HAPO-Afro-Caribbean (n=1224)			HAPO-White (n=7679)		
	β (95% CI)	P value	R^2^	β (95% CI)	P value	R^2^	β (95% CI)	P value	R^2^
Fasting glucose (mmol/L)	104 (58.6 to 149)	<0.001	0.008	203 (137 to 270)	<0.001	0.036	239 (214 to 265)	<0.001	0.043
1-hour glucose (mmol/L)	29.4 (15.3 to 43.5)	<0.001	0.007	45.1 (28.4 to 61.7)	<0.001	0.020	45.8 (40.0 to 51.1)	<0.001	0.030
2-hour glucose (mmol/L)	33.2 (15.0 to 51.4)	<0.001	0.005	56.2 (35.6 to 76.8)	<0.001	0.020	57.7 (50.0 to 65.3)	<0.001	0.030
Maternal age (years)	6.0 (2.3 to 9.7)	0.001	0.004	10.7 (6.4 to 15.0)	<0.001	0.010	8.34 (6.6 to 10.1)	<0.001	0.012
Maternal BMI	10.7 (7.8 to 13.6)	<0.001	0.020	13.7 (9.7 to 17.7)	<0.001	0.050	20.2 (18.3 to 22.1)	<0.001	0.055
Parity	88.3 (46.5 to 130)	<0.001	0.006	85.8 (31.9 to 140)	<0.001	0.007	117 (95.3 to 138)	<0.001	0.015
Systolic BP (mm Hg)	0.53 (−1.45 to 2.50)	0.601	0.000	3.32 (0.87 to 5.76)	0.008	0.006	4.49 (3.53 to 5.44)	<0.001	0.011

Birth weight corrected for sex and gestational age. β, beta coefficient represents the increase in birth weight, in grams, for a 1 SD increase in the predictor; statistical univariate analysis.

BMI, body mass index; BP, blood pressure; HAPO, Hyperglycaemia and Adverse Pregnancy Outcome Study.

### The relationship between maternal glycaemia and adverse pregnancy outcome of LGA (birth weight > 90th percentile) is weaker in Uganda compared with other cohorts

Table 3 shows the association of maternal glucose as a continuous variable with LGA, including adjusted ORs and 95% CIs for each 1 SD higher glucose concentration with adjustments for confounders as in HAPO. An increase in glucose by 1 SD was associated with an increased odds of LGA in all the studies. However, the effect was smaller in Uganda compared with the other studies (adjusted OR (95% CI) 1.13 (1.00 to 1.29) in Uganda, 1.38 (1.15 to 1.66) HAPO-Afro-Caribbean and 1.57 (1.46 to 1.69) HAPO-white).

**Table 3:** Adjusted OR for the association between maternal glycaemia as a continuous variable and large for gestational age (LGA) (corrected birth weight >90th percentile)

	Uganda (n=2544)		HAPO-Afro-Caribbean (n=1224)		HAPO-White (n=7679)	
	AOR (95% CI)	R^2^	AOR (95% CI)	R^2^	AOR (95% CI)	R^2^
Fasting glucose	1.13 (1.00 to 1.29)	0.0023	1.38 (1.15 to 1.66)	0.0152	1.57 (1.46 to 1.69)	0.0297
1-hour glucose	1.21 (1.07 to 1.37)	0.0052	1.23 (1.03 to 1.48)	0.0064	1.45 (1.34 to 1.56)	0.0198
2-hour glucose	1.12 (0.99 to 0.27)	0.0018	1.19 (1.00 to 1.42)	0.0045	1.37 (1.28 to 1.47)	0.0150

ORs are for LGA per a 1 SD higher maternal fasting plasma glucose in the individual studies.

AOR, adjusted OR; HAPO, Hyperglycaemia and Adverse Pregnancy Outcome Study.

### Weaker relationship between maternal post-load glucose concentration and corrected fetal birth weight and LGA in Uganda compared with the other studies

We conducted the same analysis using 1-hour and 2-hour post-glucose load comparing Uganda with the HAPO-Afro-Caribbean and white groups. Similar to fasting glucose, 1-hour and 2-hour glucose had a weaker relationship with birth weight in Uganda than in HAPO-Afro-Caribbean and HAPO-white (online supplemental figure 1 and table 2). However, for LGA, the Uganda population had a reduced association of 1 and 2 hours post-OGTT glucose with LGA compared with the white HAPO cohort, but the relationship was broadly similar between Uganda and the Afro-Caribbean HAPO cohort (though CIs for this outcome were wide). Similar findings were observed when adjusted for maternal BMI and age at the time of OGTT (online supplemental table 3).

## Discussion

By comparing three birth cohorts, we have shown that the maternal fasting and post OGTT glucose contributions to fetal birth weight are substantially lower in women in Uganda than in women in the HAPO study despite having similar BMI. We also found that an increase of glucose by 1 SD in Ugandan women was associated with a smaller OR of having an LGA baby than in the other populations. Taken together, this suggests that glucose cut-offs for diagnosis of gestational diabetes need to be population-based, and thresholds derived from high and middle income countries participating in HAPO may not be appropriate for use in SSA.

### Possible explanations for the lower contributions of maternal glucose to birth weight in Uganda

Many factors influence birth weight, including infant sex, gestational duration, maternal nutrition, ethnicity, parity and the mother’s weight and structure.^10^ The maternal glucose concentration is a key determinant, as glucose is the primary substrate for fetal growth. It is transported across the placenta proportionally to its concentration and stimulates fetal insulin and insulin-like growth factors.^11^ This physiological link explains why increased maternal glucose concentrations, often due to increased insulin resistance, facilitate greater transplacental glucose transfer, leading to excess fetal fat storage and, consequently, a greater risk of LGA (macrosomia).^12^ However, it is crucial to recognise that in all populations, maternal glucose explains only a small fraction of the total variance in birth weight.

The reasons maternal glycaemia might have a smaller impact on birth weight in SSA are complex and likely involve multiple competing factors that may override or dampen the glucose-driven anabolic signal. In SSA, fetal growth might be influenced by non-glucose growth constraints, for example, nutrition and environment. The high burden of maternal undernutrition and micronutrient deficiencies in this region may impose nutritional constraints on fetal development that glucose availability alone cannot overcome.^13^ Specifically, if other critical nutrients are lacking, the effect of high glucose on increasing birth weight is blunted. For example, global studies consistently correlate maternal milk intake with higher gestational, placenta and fetal birth weight, and SSA region has the lowest maternal milk consumption in the world.^14^,^16^

Differences in the placenta itself may mediate the weaker association. This might be due to underlying genetic factors, poor growth environments or, critically, infections. A higher prevalence of infections in SSA, such as malaria and HIV/AIDS, can lead to placental inflammation and insufficiency. This damage restricts the transplacental transfer of all nutrients, including glucose, regardless of the maternal concentration, thereby directly contributing to the weaker glucose-birth weight relationship observed in our study.

Differences in overall glucose metabolism are also relevant. In our study, we found that women in Uganda had lower post-challenge glucose concentrations than white women despite comparable fasting glucose levels, suggesting upregulated beta-cell function. Available data similarly suggest that black individuals tend to exhibit greater insulin resistance and coupled with upregulated beta-cell function compared with white populations.^17^ The comparable findings between the Uganda and Afro-Caribbean HAPO participants, in our analysis, provide potential insights into the genetic and metabolic mechanisms accounting for these ethnicity-specific differences in the relationship between glucose and fetal growth.

However, many of these proposed mechanisms could not be directly assessed in this study and remain important evidence gaps requiring further targeted interventions.

### The financial implications of universal screening and using lower cut-offs to diagnose GDM in SSA need to be debated

Our study has a number of implications. First, glycaemic thresholds of risk for adverse pregnancy outcomes may be higher in SSA compared with high income countries. This suggests that it may be necessary to revise the international guidelines to make them appropriate for SSA populations. This may avoid unnecessary intervention in those who would otherwise be considered at risk with international guidelines, but not with SSA specific guidelines. There are notable differences in the epidemiology of type 2 diabetes and GDM in terms of ethnicity; black women have a higher prevalence of type 2 diabetes but not of GDM when compared with white women.^18^,^20^ So, therefore, identification of GDM in pregnancy might not be a marker of future type 2 diabetes risk.

Concerns have been raised on the cost implications of the IADPSG guidelines that recommend lower FPG cut-off of 5.1 mmol/L for diagnosis of GDM. It has been demonstrated that the IADPSG criteria increase GDM diagnosis by almost two-fold in some populations, and this has drastic economic implications that impact the utility of this test, especially in low resource settings.^21^ The guidelines also recommend that all pregnant women should be tested for GDM (universal screening).^2^ However, universal screening for hyperglycaemia during pregnancy is not practical in many low-resource settings, including those in SSA.^22^ Many healthcare systems in Africa employ a selective screening approach for GDM whereby only women with certain risk factors are tested.^23^ This approach is viewed as the most cost-effective in resource-poor settings.^24^ However, the disadvantage of selective screening is the lack of data on the actual prevalence of the condition and also the risk of affected women being missed; selective screening is thought to miss around 50% of women with GDM.^25^

Our study found similar results using fasting and post glucose load concentrations. The gold-standard test for diagnosing GDM is the OGTT performed at 24–28 weeks gestation.^26^ In resource-poor settings where OGTTs are not feasible, a FPG screen may be an alternative option for detecting women with GDM. There has been some debate around whether FPG reading alone is sufficient for diagnosing GDM^27 28^ and predicting adverse neonatal outcomes.^29^ FPG reading alone appears to have a high sensitivity in detecting GDM in SSA.^30 31^ In a study involving 1906 pregnant women in South Africa, FPG reading had a high sensitivity (83.3% (95% CI 77.0% to 88.5%) in diagnosing GDM.^30^ A more recent analysis of the HAPO Study also found that using FPG for detection of GDM appears to identify a large majority of GDM cases while eliminating the need to perform a full OGTT in more than half of the participants.^32^

### Strengths and weaknesses

The contribution of gestational diabetes to maternal and neonatal adverse outcomes in SSA is not well documented.^33^ A strength of this study is that it compares a large, well-characterised population in Africa with the HAPO Study that was used to determine the diagnostic criteria for GDM. Limitations are that the study only represents findings from one country in SSA, and it is unclear whether the results can be generalised to the entire continent. A further limitation is that there were limited data available to enable exploration of factors that might further explain the differences observed, for example, maternal and cord C-peptide to compare levels of insulin secretion and resistance between the populations. Approximately 30% of participants were excluded in the analysis due to incomplete neonatal data. Although the excluded women were younger, their glucose levels were similar to those included, suggesting limited selection bias. However, this may still affect representativeness and should be considered when interpreting the findings. FPG was measured at an earlier gestational age in Uganda than in the HAPO Study. Our study did not look at other adverse effects attributed to GDM, for example, caesarean section. However, other predictors for a caesarean section have been shown to play a crucial role in deciding to conduct a caesarean section in many SSA and other regions. Cultural preference seems to play an important role in the caesarean section rate.^34^

## Conclusions

The contribution of maternal glucose to birth weight and the risk of LGA at a given level of hyperglycaemia is significantly lower in SSA than in populations in the HAPO study. These data support further validation across diverse SSA populations and may ultimately inform the development of locally appropriate glycaemia cut-offs to identify women at risk of adverse pregnancy outcomes.

## Supplementary material

10.1136/bmjgh-2025-019569 online supplemental file 1

10.1136/bmjgh-2025-019569 online supplemental file 2

10.1136/bmjgh-2025-019569 online supplemental figure 1

## Data Availability

Data are available on reasonable request.
