# Supplementary material for: The contribution of maternal glucose to birth weight is smaller in Uganda (sub-Saharan Africa) than in Afro-Caribbean or white ethnicity mother–child pairs from outside Africa
Source: BMJ Glob Health. 2026 Mar 9;11(3):e019569. doi: 10.1136/bmjgh-2025-019569 (PMC12983726; doi:10.1136/bmjgh-2025-019569)
Supplement: online supplemental file 1 [file bmjgh-11-3-s001.docx]

**Supplementary Data**

**Table of Contents**

[Table 1 Comparison of participants excluded because of incomplete data with those included in the analysis. 2](#_Toc183043325)

[Table 2 Sensitivity analysis comparing the association between adjusted birth weight and maternal glycaemia and other maternal characteristics in Uganda excluding women with fasting glucose >5·8 mmol/L as in the HAPO study or fasting glucose >5·1 mmol/L (the threshold recommended by the IADPSG for diagnosis of gestational diabetes mellitus). 3](#_Toc183043327)

[Table 3 Adjusted Odds Ratio for the association between maternal glycaemia and Large for Gestational Age (Corrected Birthweight >90th Percentile) adjusted for maternal age, BMI and parity. 4](#_Toc183043326)

**Table 1 Comparison of participants excluded because of incomplete data with those included in the analysis.** Mean ±SD for continuous variables n (%) for proportions

| Variable | With Missing data  N=974 | With Complete data  N=2657 | P-Value |
| --- | --- | --- | --- |
| Mothers age (yrs) | 25·7±5·5 | 27·0±5·5 | <0·001 |
| BMI (kg/m2) | 27·2±4·9 | 27·8±6·8 | 0·011 |
| Systolic BP (mmHg) | 104±10·6 | 104±10·1 | 0·023 |
| Fasting glucose (mmol/L) | 4·3±0·6 | 4.3±0·7 | 0·16 |
| 1-hour (mmol/L) | 6·3±1·6 | 6·4±1·6 | 0·57 |
| 2-hour (mmol/L) | 5·8±1·5 | 5·9±1·4 | 0·41 |
| Length of gestation at time of OGTT (weeks) | 25·8±1·2 | 25·9±1·2 | 0·002 |

**Table 2 Sensitivity analysis comparing the association between adjusted birth weight and maternal glycaemia and other maternal characteristics in Uganda excluding women with fasting glucose >5·8 mmol/L as in the HAPO study or fasting** **glucose >5·1 mmol/L (the threshold recommended by the IADPSG for diagnosis of gestational diabetes mellitus).**

|  | HAPO Classification:  Fasting Plasma Glucose >5·8 mmol/L (n=2544) | | IADPSG Classification  Fasting Plasma Glucose >5·1 mmol/L  (n=2463) | |
| --- | --- | --- | --- | --- |
|  | **β(95% CI)** | **R^2^** | **β(95% CI)** | **R^2^** |
| Fasting glucose (mmol/L) | 104 (58·6 – 149) | 0·008 | 111 (56·4 – 165) | 0·007 |
| 1-hr glucose (mmol/L) | 29·4 (15·3 – 43·5) | 0·007 | 30·2 (14·1 – 46·3) | 0·006 |
| 2-hr glucose (mmol/L) | 33·2 (15·0 – 51·4) | 0·005 | 32.0 (10·6 – 53·3) | 0·004 |
| Maternal Age (yrs) | 6·0 (2·3 – 9·7) | 0·004 | 5·47 (1·60 – 9·34) | 0·003 |
| Maternal BMI (kg/m^2^) | 10·7 (7·8 – 13·6) | 0·020 | 10·7 (7·66 – 13·7) | 0·020 |

**Table 3 Adjusted Odds Ratio for the association between maternal glycaemia and Large for Gestational Age (Corrected Birthweight >90th Percentile) adjusted for maternal age, BMI and parity.** As in the HAPO analysis 1, all models are adjusted for maternal BMI and age at the time of the OGTT. Odds ratios were the increase in LGA for an increase in the glucose level of 1 SD in the individual studies. AOR, Adjusted Odds Ratio; CI, confidence interval; HAPO, Hyperglycaemia and Adverse Pregnancy Outcome Study;

|  | Uganda (n=2544) | | HAPO-Afro-Caribbean (n=1224) | | HAPO-White (n=7679) | |
| --- | --- | --- | --- | --- | --- | --- |
|  | **AOR (95% CI)** | **R-squared** | **AOR (95% CI)** | **R-squared** | **AOR (95% CI)** | **R-squared** |
| **Fasting glucose** | 1·07 (0·94 – 1·23) | 0·009 | 1·29 (1·22 – 1·38) | 0·045 | 1·34 (1·24 – 1·45) | 0·053 |
| **1-hr glucose** | 1·15 (1·01 – 1·32) | 0·012 | 1·11 (0·97 – 1·35) | 0·018 | 1·29 (1·20 – 1·40) | 0·051 |
| **2-hr glucose** | 1·07 (0.94 – 1·22) | 0·009 | 1·07 (0·88 – 1·30) | 0·018 | 1·25 (1·15 – 1·34) | 0·050 |
