# Supplementary material for: The contribution of maternal glucose to birth weight is smaller in Uganda (sub-Saharan Africa) than in Afro-Caribbean or white ethnicity mother–child pairs from outside Africa
Source: BMJ Glob Health. 2026 Mar 9;11(3):e019569. doi: 10.1136/bmjgh-2025-019569 (PMC12983726; doi:10.1136/bmjgh-2025-019569)
Supplement: online supplemental file 2 [file bmjgh-11-3-s002.docx]

### BMJ Global Health Author Reflexivity Statement

Adapted from Morton, B., Vercueil, A., Masekela, R., Heinz, E., Reimer, L., Saleh, S., Kalinga, C., Seekles, M., Biccard, B., Chakaya, J., Abimbola, S., Obasi, A. and Oriyo, N. (2022), Consensus statement on measures to promote equitable authorship in the publication of research from international partnerships. Anaesthesia, 77: 264-276. <https://doi.org/10.1111/anae.15597>

| **Study conceptualisation** | |
| --- | --- |
| 1. How does this study address local research and policy priorities? | This study provides critical evidence that the impact of maternal glucose on fetal overgrowth is significantly weaker in a Ugandan population than in the Afro-Caribbean and White cohorts of the global HAPO study. Specifically, a given increase in blood glucose led to a smaller rise in birth weight and a lower risk of a large-for-gestational-age baby in Uganda.   - - - 1. Fills a Major Data Gap: It generates the first large-scale evidence from sub-Saharan Africa on this relationship, correcting the exclusion of this region from prior global studies.       2. Challenges Global Standards: It demonstrates that applying internationally derived glucose thresholds in sub-Saharan Africa is inappropriate, as they would likely lead to over-diagnosis and misallocation of scarce healthcare resources.       3. Calls for Local Action: The findings directly support the urgent need to develop and implement locally derived, evidence-based diagnostic criteria for hyperglycemia in pregnancy within sub-Saharan Africa. |
| 1. How were local researchers involved in study design? | This study was conceived and led by researchers based in Uganda, with local investigators centrally involved in defining the research questions, study design, and analytic approach. The research was conducted through an equitable partnership between the Medical Research Council/Uganda Virus Research Institute and LSHTM Uganda Research Unit, Entebbe, and the University of Exeter. Decisions regarding data analysis, interpretation of findings, and manuscript preparation were made collaboratively, ensuring that local contextual knowledge informed all stages of the research. This approach aimed to promote shared ownership, capacity strengthening, and balanced representation in the generation and dissemination of evidence. |
| **Research management** | |
| 1. How has funding been used to support the local research team(s)? | This study was funded by the UK National Institute for Health Research (NIHR; award references 17/63/131 and NIHR156184) through UK international development funding. The funding directly supported the local research team in Uganda, including the appointment of a full-time early-career researcher based at the Medical Research Council/Uganda Virus Research Institute and the LSHTM Uganda Research Unit, Entebbe. Additional support facilitated collaborative analysis and mentorship with partners at the University of Exeter, contributing to capacity strengthening and equitable research partnerships. |
| **Data acquisition and analysis** | |
| 1. How are research staff who conducted data collection acknowledged? | This analysis was conducted using existing quantitative datasets, and no new primary data collection was undertaken for this study. The research teams responsible for the original data collection are acknowledged through authorship and institutional recognition associated with the parent studies from which these data were derived. |
| 1. How have members of the research partnership been provided with access to study data? | All members of the partnership have access to data |
| 1. How were data used to develop analytical skills within the partnership? | The study provided opportunities for research fellows based at the Medical Research Council/Uganda Virus Research Institute and the LSHTM Uganda Research Unit, Entebbe, to develop advanced analytical skills through hands-on analysis of large, multi-cohort datasets, supported by mentorship from partners at the University of Exeter. The work also formed part of a PhD research project led by a jointly affiliated research fellow, focusing on evaluating the accuracy and utility of oral glucose tolerance testing in sub-Saharan Africa, thereby strengthening analytical capacity within the partnership. |
| **Data interpretation** | |
| 1. How have research partners collaborated in interpreting study data? | Interpretation of the study data was undertaken through regular collaborative meetings involving all research partners. The study team, led by the PhD fellow and supported by supervisors from each institution, met weekly from study inception to jointly plan the study design, guide analysis, interpret findings, and agree on dissemination strategies, ensuring shared decision-making throughout the research process. |
| **Drafting and revising for intellectual content** | |
| 1. How were research partners supported to develop writing skills? | Research partners were supported to develop writing and communication skills through collaborative preparation of national and international conference presentations and manuscript drafts. The writing process was led by the first author, who is based in Africa, with iterative feedback and mentorship from co-authors across institutions. This approach enabled shared learning and strengthened skills in scientific writing and dissemination across multiple formats. |
| 1. How will research products be shared to address local needs? | Study findings are being shared through national, regional, and international dissemination platforms, including policy-relevant meetings and academic forums, to ensure relevance to local stakeholders. Reports, publications, and presentations emphasise the implications of the findings for maternal health in sub-Saharan Africa, particularly the need for further validation across diverse SSA settings and the potential to inform the development of locally appropriate glycaemia thresholds to identify women at risk of adverse pregnancy outcomes. |
| **Authorship** | |
| 1. How is the leadership, contribution and ownership of this work by LMIC researchers recognised within the authorship? | Leadership, contribution, and ownership of this work by LMIC researchers are reflected in the authorship structure. The first and senior (last) authors are based in and reside in LMICs and led the conception, analysis, interpretation, and writing of the manuscript. The author list also includes researchers from the Medical Research Council/Uganda Virus Research Institute and the LSHTM Uganda Research Unit, Entebbe, who made substantial contributions across all stages of the study, including data analysis and manuscript development. |
| 1. How have early career researchers across the partnership been included within the authorship team? | Early career researchers have been included in the authorship including WPN the first author and IS, AEH and TN |
| 1. How has gender balance been addressed within the authorship? | Four main authors (AEH, TS, RMF, BMS) are female and seven main authors (WPN, IS, RCA, WLL, AGJ, ATH, MJN) are men. |
| **Training** | |
| 1. How has the project contributed to training of LMIC researchers? | This project prioritized LMIC leadership and long-term capacity building by ensuring the study was led by researchers in Uganda. The first author, a Malawian national, utilized this research as a core component of his PhD, gaining critical academic and lead-authorship experience. Additionally, the project provided dedicated funding for Ugandan research fellows to pursue specialized professional development and technical training, fostering sustainable career growth and strengthening local research infrastructure. |
| **Infrastructure** | |
| 1. How has the project contributed to improvements in local infrastructure? | The project strengthened the local research infrastructure by establishing sustainable, long-term research partnerships and collaborative networks that extend beyond the project's duration. This framework facilitates ongoing communication and data sharing, enhancing the capacity for future collaborative studies. |
| **Governance** | |
| 1. What safeguarding procedures were used to protect local study participants and researchers? | This study adhered to rigorous safeguarding protocols approved by the Uganda Virus Research Institute (GC/127/19/04/625) and the Uganda National Council for Science and Technology (HS2340). Participant protection was ensured through a comprehensive informed consent process and the de-identification of data at the point of collection to guarantee anonymity. To protect local researchers and participants alike, the study design was developed in consultation with local stakeholders to ensure it addressed community priorities and followed culturally appropriate safety standards. All data were handled according to strict confidentiality and secure storage protocols. |
